# An experimentally validated approach to calculate the blood-brain barrier permeability of small molecules

**DOI:** 10.1038/s41598-019-42272-0

**Published:** 2019-04-16

**Authors:** Yukun Wang, Erin Gallagher, Christian Jorgensen, Evan P. Troendle, Dan Hu, Peter C. Searson, Martin. B. Ulmschneider

**Affiliations:** 1 Institute for NanoBioTechnology, Johns Hopkins University, Baltimore, Maryland USA; 2 Department of Chemistry, King’s College London, London, UK; 3 Institute of Natural Sciences and Department of Mathematics, Shanghai Jiao-Tong University, Shanghai, China; 4 Department of Materials Science and Engineering, Johns Hopkins University, Baltimore, Maryland USA

## Abstract

Drug development for the treatment of central nervous system (CNS) diseases is extremely challenging, in large part due to the difficulty in crossing the blood-brain barrier (BBB). Here we develop and experimentally validate a new *in silico* method to predict quantitatively the BBB permeability for small-molecule drugs. We show accurate prediction of solute permeabilities at physiological temperature using high-temperature unbiased atomic detail molecular dynamics simulations of spontaneous drug diffusion across BBB bilayers. These simulations provide atomic detail insights into the transport mechanisms, as well as converged kinetics and thermodynamics. The method is validated computationally against physiological temperature simulations for fast-diffusing compounds, as well as experimentally by direct determination of the compound permeabilities using a transwell assay as an *in vitro* BBB model. The overall agreement of the predicted values with both direct simulations at physiological temperatures and experimental data is excellent. This new tool has the potential to replace current semi-empirical *in silico* screening and *in vitro* permeability measurements in CNS drug discovery.

## Introduction

The systemic delivery of molecular cargo into the brain is regulated by the blood-brain barrier (BBB). This transport barrier is formed by human brain microvascular endothelial cells (hBMECs) with tight junctions that prevent paracellular transport, transporters to deliver essential nutrients to the brain, and efflux pumps to transport unwanted substrates back into circulation. Thus non-specific transport into the brain is generally limited to passive diffusion across the apical and basolateral membranes of the hBMECs that form the lumen of the cerebrovasculature. The barrier function of the BBB is critical for homeostasis, but also represents a significant roadblock in delivering drugs to the brain^1^. Consequently, many central nervous system (CNS) disorders (including depression, schizophrenia, chronic pain, and epilepsy) are difficult to treat with small molecule pharmaceuticals^1^.

Drug discovery and development for the CNS typically involves *in silico* screening followed by *in vitro* assessment of permeability and toxicology, animal studies, and human trials^2^. Commonly used *in silico* models include variations of Lipinski’s rule of five, such as the CNS multiparameter optimization (MPO) score (c.f. Fig. S1)^3,4^, that empirically assess molecular size, polarity, hydrogen bond acceptor and donor numbers, and lipophilicity^5^. While lipophilicity, dipole moment, and size are factors that influence the ability of a compound to enter the hydrophobic core of a cell membrane, the lipid bilayer environment is too chemically complex, fluid, and heterogeneous for these indicators alone to provide accurate estimates of transport kinetics. As a result of the limitations of current empirical models, brain penetration of lead compounds is screened experimentally using the transwell assay^6^. A typical workflow to assess CNS penetration of novel compounds can be to measure the passive permeability using Madin-Darby canine kidney 2 low efflux (MDCKII-LE) transwell assays or parallel artificial membrane permeability assays (PAMPAs), or to determine efflux activity using multi-drug resistance gene 1 MDCK (MDR1-MDCK) or heterogeneous human epithelial colorectal adenocarcinoma (“Caco-2”) transwell assay^6^. In the transwell assay, a confluent monolayer of endothelial cells on a porous membrane separates an apical and basolateral chamber. Following introduction of a molecule into the apical chamber, the permeability is determined from the time-dependent concentration of the solute in the basolateral chamber.

As quantitative concentration measurements can be tricky and need to be validated for each compound, the ability to accurately model the mechanism and kinetics of small molecule transport across the BBB has the potential to accelerate CNS drug discovery and development. Historically, solute permeability through membranes is described qualitatively using the homogeneous solubility-diffusion model^7^. In this model, solutes are passively transported by first partitioning into a hydrophobic phase (the membrane), subsequent diffusion across this phase, followed by re-solvation at the other side. Solute permeabilities are estimated using *P* = *DK*/2*L*, where *D* is the solute diffusion coefficient in the membrane, *K* is the solute water-to-membrane partition coefficient, and *L* is the thickness of the membrane. While this model captures the basics of transport across a hydrophobic membrane, biological lipid bilayers are chemically complex precluding quantitative prediction of permeabilities. To address this issue, methodologies were proposed that utilize atomic detail simulations, which provide a more realistic description of both the transbilayer chemical profile as well as the highly fluid nature of lipid bilayers^8^. In this model, termed the inhomogeneous solubility-diffusion model^9,10^, the solute permeability is related to an integral of depth-dependent parameters, namely the diffusion coefficient *D* and membrane partition coefficient *K*, which are calculated using umbrella sampling methods^8^. While this approach has been used to predict the membrane permeability of drug molecules^11–13^, it cannot reveal transport mechanisms or provide a trajectory of a permeating molecule across the bilayer, which are desirable information for pharmacophore optimisation. Furthermore, permeation is effectively assumed to be a monomeric and one-dimensional translational process^8,14^, which can be problematic in some cases^14^.

Here we develop and experimentally validate a different approach that accurately predicts transport kinetics and trans-bilayer free energy profiles from unbiased molecular dynamics (MD) simulations that capture multiple spontaneous solute diffusion events through lipid bilayers at atomic resolution. This equilibrium method also reveals the molecular translocation mechanism in its entirety, and without bias, and represents a major shift from existing simulation methodologies.

**Figure 1:**
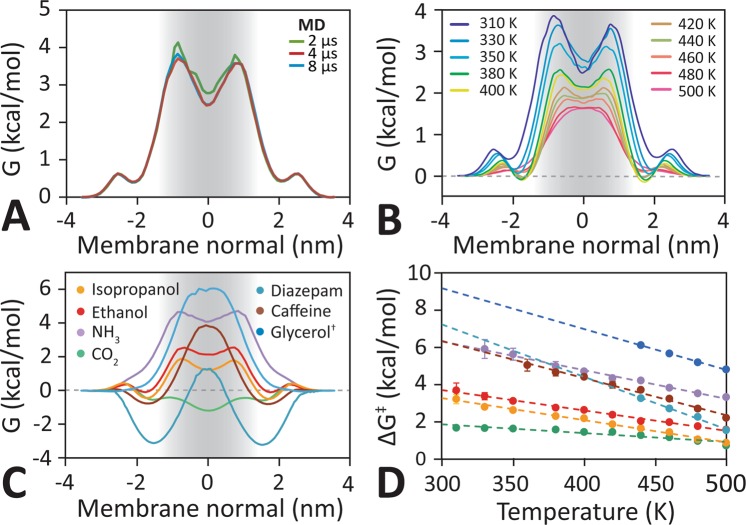
Trans-bilayer free energy barrier profile. (**A**) Free energy profiles for ethanol within the apical hBMEC lipid bilayer in 310 K obtained from unbiased equilibrium simulations with different trajectory timescales. The free energy profile converges after 4 μs. **(B**) Free energy profiles for ethanol in apical hBMEC lipid bilayers assembled from series of unbiased equilibrium molecular dynamics (MD) simulations with different temperatures ranging from 310 K to 500 K. (**C)** Glycerol and ethosuximide free energy profiles were calculated from the transmembrane solute distribution at 440 K, all other compounds were calculated at 380 K. The shape of the barrier profile is determined by the chemistry of the molecule, with no clearly recognizable pattern for small (CO_2_, NH_3_), polar (NH_3_, ethanol, isopropanol), or large (isopropanol, caffeine, ethosuximide) molecules. (**D)** The main barrier height decreases linearly with increasing temperature for all molecules. This finding allows the estimation of the barrier height using elevated temperature simulations.

Computational hardware currently limits MD simulations of the systems studied here to timescales smaller than ~100 µs, necessitating a high-temperature approach. Heating accelerates the kinetics of diffusive processes and has been successfully employed to predict membrane transfer free energies with high accuracy^15^. This method is particularly well suited for capturing membrane processes, since the changes in the chemical cross-sectional profile of lipid bilayers upon heating can be accurately predicted (see Fig. S2)^16^. Therefore, the molecular interactions of a partitioning or translocating solute with the lipid bilayer remain essentially unchanged along the transport pathway, resulting in precisely predictable changes to the transfer free energies (Fig. 1). Here we extend this approach to the extrapolation of transport kinetics. We show below that the temperature dependent deviation from perfect Arrhenius kinetics can be fully accounted for by the diffusion properties of the lipids in the membrane and are hence solute independent. This makes the method generally applicable to predict the BBB permeability of any pharmacophore that resists thermal denaturation.

## Methods

### Transbilayer solute diffusion simulations

Unbiased atomic detail MD simulations were performed using GROningen MAchine for Chemical Simulations (GROMACS) (www.gromacs.org)^17^, in combination with the Chemistry at HARvard Macromolecular Mechanics (CHARMM)^18^ general force field (CGenFF)^19^ for molecular solutes and the transferable intermolecular potential with 3 points (TIP3P) water model as solvent^20^, Lipids parameters were taken from the CHARMM36 all-atom force field^21^. Electrostatic interactions were computed using particle-mesh-Ewald (PME)^22^, and a cut-off of 10 Å was used for the van der Waals interactions. Bonds involving hydrogen atoms were restrained using LINear Constraint Solver (LINCS)^23^ to allow a 2 fs time-step. Neighbour lists were updated every 5 steps. All simulations were performed in the constant number, pressure, and temperature (NPT) ensemble, with water, lipids, and drug molecules coupled separately to a heat bath with temperatures in the range of 37–227 °C using a time constant τ_T_ = 0.1 ps in combination with the velocity rescaling algorithm^24^. Atmospheric pressure of 1 bar was maintained using Parrinello−Rahman semi-isotropic pressure coupling with compressibility κ_z_ = κ_xy_ = 4.6 · 10^−5^ bar^−1^ and time constant τ_P_ = 1 ps^25^. In order to capture diffusion events at sufficiently high resolution, trajectories were written with 1 ps temporal resolution, resulting in 1 × 10^6^ observation points for a 1 μs trajectory.

### *In vitro* solute permeability assay

To validate results from MD simulations, permeabilities for selected molecules were determined using the transwell assay with human induced pluripotent stem cell (iPSC)-derived hBMECs. Details of the experimental procedures have been reported elsewhere^26–29^. Briefly, hBMECs were sub-cultured onto polyester 12-well transwell inserts with a 0.4 µm pore size (Corning) coated with 1:1 collagen IV (100 µg mL^−1^, Sigma) and fibronectin (50 µg mL^−1^, Sigma) at a density of 1 × 10^6^ cells mL^−1^ and allowed to reach confluence for another two days. Permeability experiments were performed only if the transendothelial electrical resistance (TEER) was in excess of 1500 Ω cm^2^. Prior to permeability experiments, cells were washed with transport buffer (0.12 M NaCl, 25 mM NaHCO_3_, 3 mM KCl, 2 mM MgSO_4_, 2 mM CaCl_2_, 0.4 mM K_2_HPO_4_, 1 mM 4-(2-hydroxyethyl)-1-piperazineethanesulfonic acid (HEPES), and 0.1% human platelet poor derived serum) and 100 µL of the molecule of interest in transport buffer was introduced into the apical well. The concentration of caffeine was measured using high-performance liquid chromatography.

## Results and Discussion

### Atomic detail models of BBB bilayers

Atomic detail molecular models of the apical and basolateral lipid bilayer of hBMECs were constructed by closely replicating physiological lipid compositions (Fig. S2)^2^. The total number of lipids was 96, distributed for the apical hBMEC as N-oleoyl-d-erythro-sphingosylphosphorylcholine) (OSM) (18), cholesterol (CHOL) (28), 1-palmitoyl-2-oleoyl-sn-glycero-3-phosphocholine (POPC) (4), 1-stearoyl-2-arachidonoyl-sn-glycero-3-phosphocholine (SAPC) (8), 1-stearoyl-2-arachidonoyl-sn-glycero-3-phosphoethanolamine(SAPE) (14), 1-stearoyl-2-oleoyl-sn-glycero-3-phosphoethanolamine (SOPE) (6), 1-stearoyl-2-arachidonoyl-sn-glycero-3-phospho-L-serine (SAPS) (8), 1-stearoyl-2-linoleoyl-sn-glycero-3-phosphocholine (SLPC) (8), 1-stearoyl-2-arachidonoyl-sn-glycero-3-phosphoinositol (SAPI) (2), and for the basolateral hBMEC bilayer as OSM (22), CHOL (28), POPC (2), SAPC (6), SAPE (14), SOPE (6), SAPS (10), SLPC (6), SAPI (2). The number of water molecules was 2950. A 150 mM background NaCl ion concentration was applied to the solvated membrane, corresponding to 15 Na^+^ ions and 5 Cl^−^ ions. Both the average length of the hydrocarbon chain (18.4 carbon atoms) and number of double bonds (1.5) of the fatty acid tails were matched to equivalent experimental values (18.4 and 1.5, respectively)^3^. Each bilayer model was equilibrated for >100 ns at a range of temperatures from 310K–500K to determine the effect of heating on the time-averaged position of the principal chemical groups (CH_3_ = methyl, CH_2_ = acyl tails, P/C = phosphocholine headgroups, G/C = glycerol-carbonyl linker, H_2_O = water) along the membrane normal. Figure S1 shows that apical and basolateral bilayers have essentially identical trans-bilayer density profiles. Unlike pure POPC bilayers, that are commonly used as mimics for cellular bilayers, the acyl chain profiles are flat across much of the membrane due to the increased lipid tail order. Atomic density curves reveal the interfacial positions of the phosphates, glycerol-carbonyl, and choline groups along the membrane normal. Increasing the temperature results in both a broadening of these atomic density curves, as well as a linear shift in position towards the centre of the membrane, indicating a thinning of the membrane. However, the trans-membrane chemical profile is maintained for the entire temperature range and no membrane defects are observed.

### Compound selection

The goal of this work is to develop an *in silico* tool for rapid assessment of solute transport across lipid bilayers of the BBB. To validate this methodology we choose a number of well characterized small molecules of varying size, polarity, and chemical complexity (see Table S1). Carbon dioxide (CO_2_), ammonia (NH_3_), ethanol (C_2_H_5_OH), and isopropanol (C_3_H_7_OH) are small molecules that are prevalent in biological systems and readily permeate lipid bilayers in sub-microsecond timescales^30^. This allows us to capture a sufficient number of spontaneous transport events to fully characterize their transport mechanisms, kinetics, and thermodynamics via multi-microsecond equilibrium MD simulations at physiological temperatures. However, diffusion of these small molecules across lipid bilayers is too rapid to allow accurate experimental quantification^31^. Caffeine is slightly larger and known to permeate the BBB with moderately high permeability^32^, whereas the anti-seizure drug ethosuximide and the polar metabolite glycerol have relatively low permeability^32,33^. Together these compounds span five orders of magnitude in experimentally determined permeability. All of these molecules passively diffuse across membranes and are not substrates of the P-glycoprotein efflux pump^32^. This allows direct comparison of computationally and experimentally determined transbilayer permeabilities. Optimized force field parameters were taken from the CHARMM generalized force field^19^ for carbon dioxide, ethanol, isopropanol, ethosuximide and glycerol. Newly-developed CHARMM parameters were taken for caffeine^34^.

### Deriving trans-bilayer free energy profiles from spontaneously diffusing solutes

Trans-bilayer Gibbs free energy profiles are calculated from the simulation-averaged population density *ρ*(*z*) along the membrane normal *z*, using *G*(*z*) = −*k*_B_*T* ln(*ρ*(*z*)), where *k*_B_ is the Boltzmann constant and *T* is the temperature (see Fig. S3). A bin width of 1 Å was chosen and errors were calculated using block-averaging with temporal blocks of 100 ns. The translocation barrier height, Δ*G*^‡^, is calculated by averaging and subtracting the value of the two lowest free energy minima (*G*_A_ and *G*_C_ in Fig. S4) on either side of the maximum barrier *G*_*B*_.

**Figure 2:**
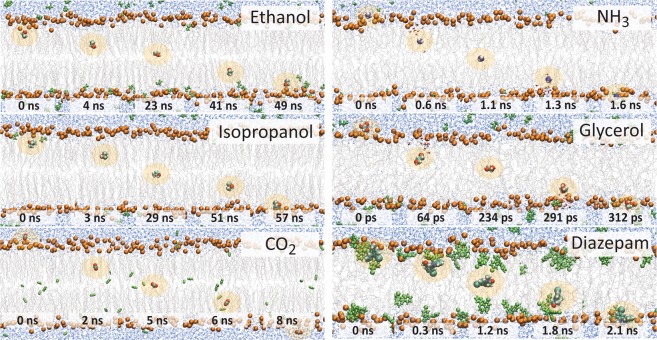
Spontaneous diffusion mechanism of small solutes and drugs through the apical BBB bilayer. Snapshots of spontaneous diffusion process of ethanol, isopropanol, and CO_2_ at a temperature of 310 K, as well as NH_3_, glycerol, and ethosuximide at 440 K. Ethanol, isopropanol, and ethosuximide directly partition into and permeate across the lipid bilayer without a water shell. In contrast, the more polar molecules NH_3_ and glycerol pull their hydration shell into the hydrophobic part of the membrane and only lose their accompanying water molecules near the centre of the membrane. The hydrophobic molecule CO_2_ prefers to stay partitioned inside of the bilayer.

In the simulations, an apical or basolateral membrane model is placed into a rectangular cuboid box containing water molecules. Periodic boundary conditions are applied such that a molecule exiting the box through one face will re-enter through the opposing face. All solute molecules are initially placed in water and are allowed to diffuse freely into, out of, and across the lipid bilayer. A summary of the systems and simulation parameters is given in Table 1. Figure 1A shows that 4 µs captures a sufficient number of spontaneous ethanol transport events (5) at 310 K to provide a converged trans-bilayer free energy barrier profile. This free energy profile has two maxima, symmetrically located near the glycerol-carbonyl region of the lipids, with a maximal barrier height of Δ*G*^‡^ = 3.6 ± 0.4 kcal/mol. Extending this simulation to 10 μs, which takes over two months to run on current high-end hardware, captures 11 ethanol translocation events at 310 K (see Fig. S5) and results in an identical free energy profile. In Fig. 2 we compare individual permeation events at 310 K and 440 K, respectively. We find that at 310 K, a single permeation event occurs on a timescale of ∼10^1^ ns, while at 440 K the timescale is ∼10^−1^ to ∼10^0^ ns. At 310 K, CO_2_ permeates ∼8-fold times faster than either ethanol or isopropanol, while at 440 K, glycerol is ∼4 fold times faster than NH_3_ and ∼7 faster than ethosuximide.

**Table 1:** Summary of the unbiased simulations listing the simulation length (*t*) and number of spontaneous transitions (*N*_trans_).

T [K]	CO_2_^†^		NH_3_^†^		Iso-propanol^†^		Ethanol^‡^		Caffeine^‡^		Glycerol^‡^		Etho-suximide^‡^		Membrane	
	*t* [μs]	*N*_trans_ [#]	*t* [μs]	*N*_trans_ [#]	*T* [μs]	*N*_trans_ [#]	*t* [μs]	*N*_trans_ [#]	*t* [μs]	*N*_trans_ [#]	*t* [μs]	*N*_trans_ [#]	*t* [μs]	*N*_trans_ [#]	A^*^ [nm^2^]	*d*_P-P_^$^ [nm]
310^**a**^	4.0	283	4.0	0	4.0	5	10	11	—	—	—	—	—	—	0.453	4.86
330^**a**^	4.0	956	4.0	1	4.0	31	3.0	25	8.6	1	—	—	—	—	0.492	4.64
350^**a**^	2.0	940	2.0	3	2.0	67	2.0	76	—	—	—	—	—	—	0.523	4.46
350^**b**^	—	—	—	—	—	—	1.3	47	—	—	—	—	—	—	0.524	4.44
360^**a**^	—	—	—	—	—	—	—	—	4.0	15	—	—	—	—	0.546	4.36
380^**a**^	1.0	1010	1.0	14	1.0	146	1.0	188	4.0	33	—	—	—	—	0.573	4.16
380^**b**^	—	—	—	—	—	—	0.6	118	—	—	—	—	—	—	0.574	4.17
400^**a**^	0.5	784	0.5	21	0.5	136	0.6	244	1.4	46	—	—	—	—	0.606	4.02
400^**b**^	—	—	—	—	—	—	0.5	209	1.0	30	—	—	—	—	0.608	3.98
420^**a**^	0.2	448	0.2	28	0.2	102	0.2	159	1.0	85	—	—	—	—	0.639	3.84
420^**b**^	—	—	—	—	—	—	—	—	1.0	69	—	—	—	—	0.638	3.81
440^**a**^	0.2	869	0.2	48	0.2	192	0.2	323	1.0	147	2.0	19	0.4	32	0.671	3.70
440^**b**^	—	—	—	—	—	—	—	—	1.0	177	—	—	—	—	0.669	3.67
460^**a**^	0.2	1985	0.2	114	0.2	266	0.2	474	0.32	120	2.0	50	0.4	55	0.703	3.64
480^**a**^	0.2	3410	0.2	210	0.2	458	0.2	816	0.32	253	2.0	129	0.4	118	0.739	3.52
500^**a**^	0.2	5265	0.2	351	0.2	637	0.2	1375	0.32	611	1.0	130	0.3	160	0.772	3.44

^†^Aqueous solute concentration = 182 mM (10 molecules per system). ^**‡**^Aqueous solute concentration = 410 mM (20 molecules per system). ^**a**^Apical lipid bilayer. ^**b**^Basolateral lipid bilayer. *A = average area per lipid. ^$^*d*_P-P_ = Average distance between the phosphates in the upper and lower bilayer leaflet (c.f. Fig. S1).

### Transbilayer free energy profiles for low permeability compounds

Table 1 shows that for larger or more polar molecules, such as caffeine, glycerol, and NH_3_, no transport events through lipid bilayers are observed in multi-μs timescale simulations at 310 K, precluding calculation of a transbilayer free energy profile. Capturing transport events for these molecules would require atomic detail multi-millisecond simulations, which are unfeasible on current hardware. Instead, we elevate the simulation temperature, which accelerates the rate of passive trans-bilayer diffusion. Figure 1B shows that the temperature increase is concomitant with a change in the trans-bilayer free energy profile and results in a flattening of the two interfacial maxima, which fuse into a single central energy barrier at very high temperatures (T > 480 K). Despite this change in barrier profile topology Fig. 1C demonstrates that the barrier maximum, Δ*G*^‡^, scales perfectly linearly with temperature. This effect was observed for all molecules studied here (see Fig. S6), and Fig. 1D shows that it is independent of the overall barrier profile shape. This suggests that an accurate estimate of the free energy barrier of hBMEC bilayers at physiological temperatures can be obtained from unbiased atomic detail equilibrium simulations at elevated temperatures, even when the diffusion rates of the molecules are too small to be captured directly at low temperatures with current hardware.

The linear change of the free energy barrier for bilayer transport with temperature (Fig. 1D) can be fitted using:

1 $${\rm{\Delta }}{G}^{\ddagger }(T)={G}_{0}-g\cdot T,$$

where *G*_0_ and *g* are compound-dependent constants listed in Table S2.

Free energy profiles have a variety of different trans-bilayer shapes. Larger polar molecules like caffeine and glycerol show high energy barriers centred at the hydrophobic core of the membrane, while the much smaller polar NH_3_ shows a wide barrier with a central minimum. Amphiphilic molecules like ethanol and isopropanol also have central minima with flanking symmetric free energy barriers as well as interfacial global free energy minima. These interfacial minima show that these molecules prefer interfacial binding and are due to the molecule orienting to place their hydrophobic tails towards the hydrophobic membrane core, while pointing the alcohol groups towards the aqueous interface.

Figure 2 shows that each class of molecule studied here displays a different translocation mechanism. Amphiphilic isopropanol, ethanol, and ethosuximide show a preference for the interface and translocate by dehydrating their alcohol groups. While the more polar glycerol and NH_3_ drag water molecules into the hydrophobic core of the membrane during translocation.

### Calculating transport rates from unbiased MD simulations

The molecular rate of transport, *r*, across the bilayer is calculated from unbiased MD simulations of spontaneous trans-bilayer solute diffusion by dividing the number of transport events observed during a simulation by the total time (*r* = *#*/*t*). Transport events are captured by tracking the progress of individual molecules through planes perpendicular to the bilayer normal located at either interface. This allows differentiating molecules that diffuse through the membrane from those that pass onto the other side of the bilayer through the water of the periodic boundaries of the simulation box. The location of the planes is shown in Fig. S4.

As diffusion across a membrane is a first order process, the transport rate is proportional to the solute concentration: *r* = *k* · *C*, where *C* is the solute concentration and the constant of proportionality is the rate constant *k*. The rate constant *k* for each solute is calculated by dividing the transport rate by the number of molecules, *N*, that cross the bilayer in the simulation system, which has approximately constant volume. To compare to experimental measurements the total flux observed is divided by 2 as the simulations capture bi-directional flux, whereas the experiments measure flux in one direction only (i.e. *P*_sim_ = *k*/2). Note that we assume that transport in the cytosol is fast compared to transport across the cell membrane.

### Temperature dependence of spontaneous transport kinetics

Unbiased equilibrium simulations can provide quantitative predictions of the total solute flux across a lipid bilayer by counting the number of spontaneous transmembrane transitions. However, current hardware limits simulation times to <100 μs for the type of systems presented here. Thus, at present, sampling of transport events for molecules that have a room-temperature trans-bilayer flux smaller than ~10^3^ s^−1^ nm^−2^ is *de facto* unfeasible using equilibrium simulations. Since the spontaneous transbilayer flux increases dramatically with temperature, being able to accurately predict the flux at physiological temperatures from elevated temperature simulations is desirable.

**Figure 3:**
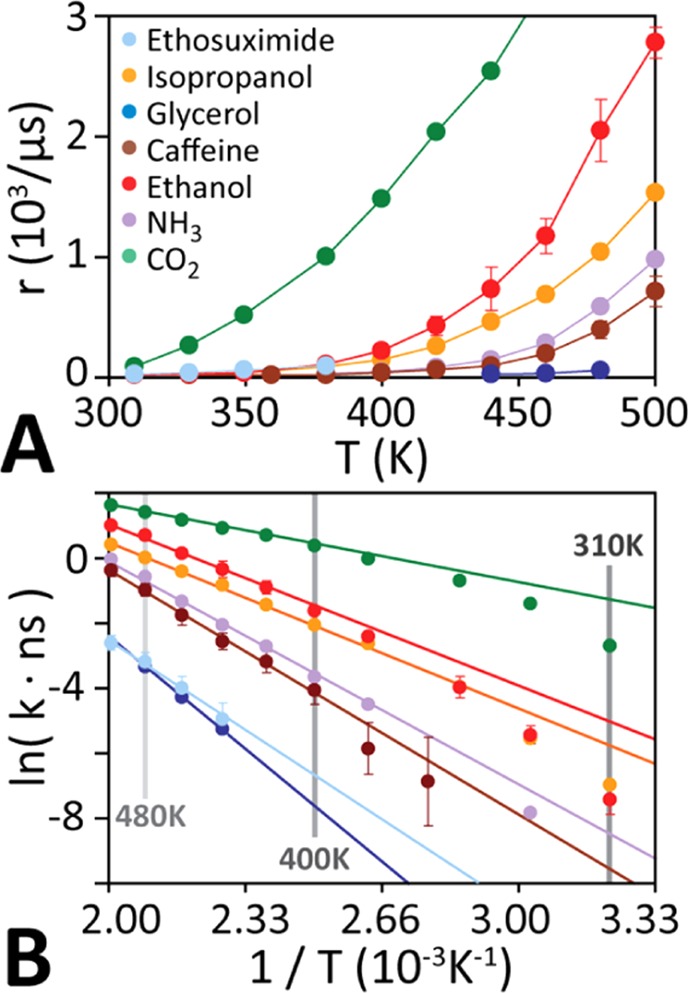
Temperature dependence of diffusion kinetics. (**A**) The spontaneous trans-bilayer transition rate increases monotonically and near-exponentially with temperature for all solutes. (**B)** Arrhenius plots of the transport kinetics obtained from multiple unbiased equilibrium simulations at different temperatures. At high temperatures, all molecules exhibit single-exponential kinetics, but some molecules deviate from Arrhenius behaviour at lower temperatures.

Figure 3A shows that the solute trans-bilayer transport rate *r* increases near-exponentially with temperature. For Arrhenius behaviour, the rate constant *k* (= *r*/*C*) decreases exponentially with the free energy barrier height Δ*G*^‡^:

2 $$k=A\cdot \exp ({\textstyle \text{-}}{\rm{\Delta }}{G}^{\ddagger }/{k}_{B}T),$$

where *k*_B_ is the Boltzmann constant, *T* is the temperature, and *A* is the attempt frequency (units s^−1^).

### Extrapolating low-temperature transport kinetics from high-temperature simulations

For ideal Arrhenius behaviour *A* is independent of temperature and Δ*G*^‡^ is either constant or varies linearly with temperature. Thus plotting ln(*k*) against 1/*T* yields a straight line with a negative slope. Figure 3B shows that at elevated temperatures (T > 400 K), the transport rate of all molecules indeed exhibits perfect Arrhenius behaviour. However, at lower temperatures (T < 400 K) all molecules show deviation from perfect single-exponential kinetics. This deviation from Arrhenius behaviour stems from the non-trivial temperature-dependence of the attempt frequency *A*(*T*) in the Arrhenius equation. This precludes a zero-error linear extrapolation of physiological-temperature kinetics from elevated temperature simulations using an Arrhenius plot. We show below that *A*(*T*) can be accurately determined from the temperature dependence of the average lateral (i.e. in the plane of the bilayer) lipid diffusion constant *D*_L_(*T*), allowing us to obtain low-temperature transport kinetics from high-temperature simulations for any solute.

Diffusive transport over a free energy barrier can be modelled using Kramer’s theory of reaction rates^35^. This theory has been successfully adapted to model micelle dissociation of amphiphilic molecules^36^ and membrane partitioning of peptides^37,38^. According to this theory the attempt frequency *A*(*T*) is related to the diffusion constant at the barrier peak:

3 $$A({T})=c\cdot {D}_{Z}({\rm{T}})/{{\rm{l}}}_{{\rm{b}}}^{2}({\rm{T}}),$$

where *l*_*b*_(*T*) is the width of the free energy barrier across the bilayer and *D*_Z_(*T*) is the diffusion constant of the diffusing molecules near the barrier peak along the bilayer normal; *c* is a temperature independent constant. *l*_*b*_^2^/*D*_Z_ has units of time and can be thought of as the time required by a single molecule to diffuse over the width of the barrier *l*_*b*_, while exp(−Δ*G*^‡^/*k*_B_*T*) in Eq. 2 can be thought of as the relative probability that a molecule resides in the region of length *l*_*b*_, or within *k*_B_*T* energy units of Δ*G*^‡^.

*D*_Z_(*T*) can be calculated using umbrella sampling. Figure S7 shows calculated solute diffusion coefficients along the membrane normal near the free energy barrier apex for H_2_O, CO_2_, NH_3_, methanol, ethanol, caffeine, and cholesterol for 10 temperatures between 310 K and 500 K for apical hBMEC lipid bilayers. Details of the calculations are given in the *Supporting Material*. As expected, *D*_Z_(*T*) increases rapidly with temperature. Since none of the solutes studied here undergo structural changes upon heating this increase in transbilayer diffusion constant must stem from the increased fluidity and decreased density of the bilayer at higher temperatures. We have previously shown that heating of a lipid bilayer from 30 °C to 180 °C does not shift the relative positions of the principal chemical groups (e.g. phosphates, glycerol-carbonyl linkers, etc.) along the membrane normal, while the bilayer density decreases overall^16^.

**Figure 4:**
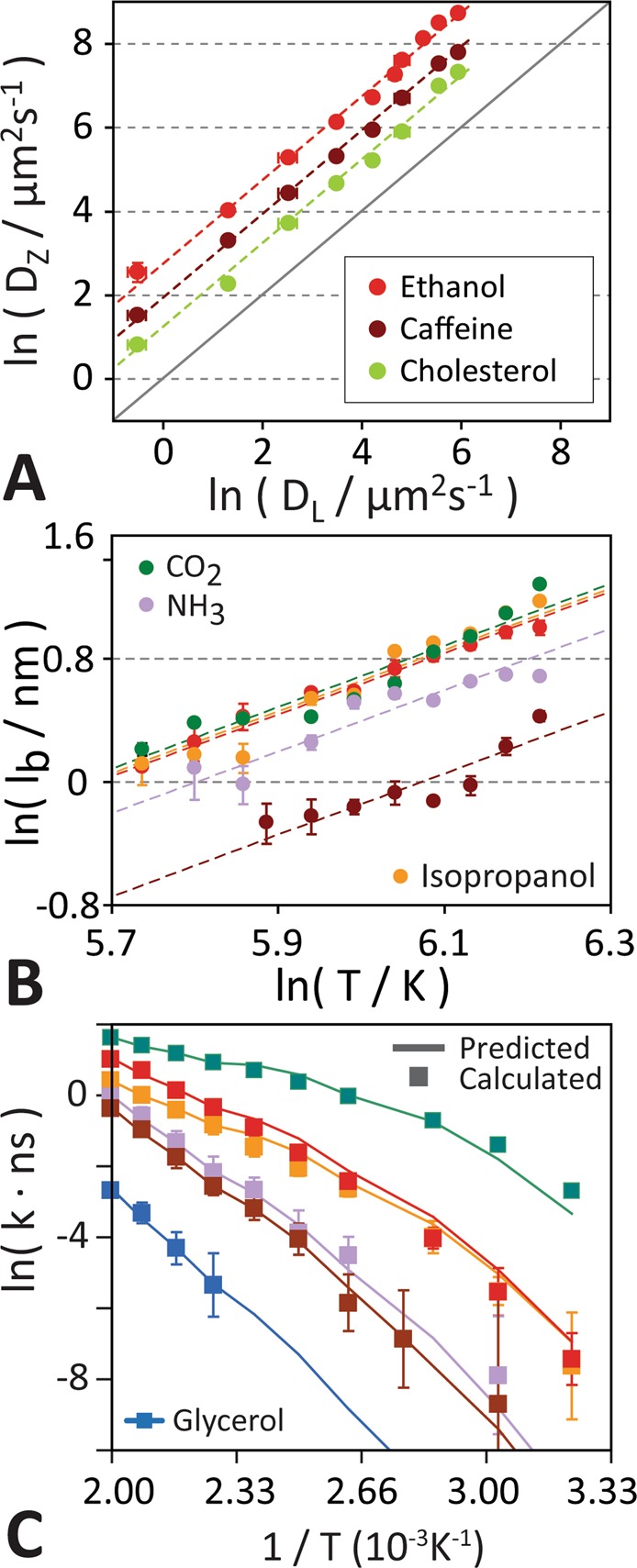
Extrapolation of low temperature kinetics from high temperature simulation. (**A**) *D*_L_ is perfectly linearly correlated with the solute diffusion coefficient at the barrier apex, *D*_Z_, for all solutes, and as a control, the lipid diffusion coefficient for cholesterol is shown for reference. (**B)** The barrier width *l*_b_ is calculated using Eq. 5. For all solutes this revealed a power law relationship between *l*_b_ and ln(T) with an average slope of 2. This means that the barrier width increases quadratically with temperature: *l*_b_(*T*) = *a* · *T*^2^, where *a* is a solute dependent constant. (**C)** Arrhenius plot, showing a quantitative comparison of predicted (line) with calculated single-molecule transport rates *k*. Predicted rates were extrapolated from fits of four high-temperature simulations (*T* = 440 K, 460 K, 480 K, and 500 K). Comparison with directly calculated rates for ethanol, isopropanol, NH_3_, CO_2_, and caffeine show excellent quantitative prediction of low-temperature kinetics.

Figure 4A shows that for all solutes the temperature dependence of the lateral (i.e. in-plane) diffusion of lipids in the apical hBMEC bilayer, *D*_L_(*T*), is perfectly correlated with the solute diffusion coefficient at the barrier apex, *D*_Z_(*T*). There are nine different lipid species in our apical bilayer model. Each of these lipid types diffuses at a different rate, thus the averaged lateral mean square displacement of the phosphate group of all the lipids was used to calculate the averaged lateral lipid diffusion coefficient. *D*_Z_(*T*) can be predicted by fitting of *D*_Z_ for 3–4 high-temperature simulations using:

4 $$\mathrm{ln}({{\rm{D}}}_{{\rm{Z}}})=\,\mathrm{ln}({\rm{b}})+{{\rm{m}}}_{{\rm{D}}}\cdot \,\mathrm{ln}({{\rm{D}}}_{{\rm{L}}})$$

We observed *m*_*D*_ = 1 for all compounds, while *b* is a compound-dependent constant that can be obtained from high-temperature simulations (*T* > 400 K). *D*_L_(*T*) is bilayer-specific and was calculated for the apical bilayer (Fig. S7). Values for *b* are given in Table S2.

Next, we need to determine the width of the free energy barrier *l*_*b*_(*T*). The definition of Aniansson:

5 $${l}_{b}={\int }_{-h}^{h}\,{e}^{\frac{G(z)-{\rm{\Delta }}{G}^{\ddagger }}{{k}_{B}T}}dz$$

relates *l*_*b*_(*T*) to *G*(*z*,*T*), the free energy profile along the membrane normal at temperature *T* and Δ*G*^‡^, the barrier height, with *h* being the half-width of the bilayer. As *G*(*z*,*T*) rapidly drops to zero outside the bilayer for all compounds the contribution to the integral outside the bilayer is negligible (see Fig. S8). For consistency between different simulations boxes, and to avoid any bias due to fluctuations in bilayer width, we choose *h* to be half the box length of the simulation (*h* ≈ 40 Å), which gives a fully converged integral for all compounds.

To obtain *l*_*b*_(*T*) from high-temperature simulations we note that free-energy barrier profiles generally broaden near the apex at high temperatures (see Figs 1B & S6). Calculation of the barrier width *l*_*b*_ for each molecule at different temperatures revealed a compound-independent power law relationship between *l*_*b*_ and temperature (Fig. 4B). Linear fitting to:

6 $$\mathrm{ln}({{l}}_{{\rm{b}}})=\,\mathrm{ln}(a)+{m}_{l}\cdot \,\mathrm{ln}(T),$$

revealed that the slope *m*_*l*_ is compound independent with an average of *m*_*l*_ ≈ 2.0 for all solutes studied here, while *a* is a compound dependent constant (Table S2). This allows determination of *l*_*b*_(*T*) from high-temperature spontaneous transbilayer diffusion simulations, akin to *D*_Z_(*T*) above.

Using Eqs 4 and 6 we are now able to determine the temperature dependence of the attempt frequency *A*(*T*) using linear extrapolation from high-temperature simulations:

7 $$A(T)=c^{\prime} \cdot {D}_{L}(T)/{T}^{4},$$

which allows us to rewrite the Arrhenius equation (Eq. 2) as:

8 $$k=c^{\prime\prime} \cdot {D}_{L}(T)/{T}^{4}\cdot \exp (\,-\,{G}_{0}/{k}_{B}T),$$

where *c*″ (= *b*/*a*^2^) and *G*_0_ are temperature independent constants that are obtained from fits to high-temperature transbilayer diffusion simulations for each compound (see Table S2).

To validate our model we calculated the transport rates for CO_2_, NH_3_, ethanol, isopropanol, and caffeine over a wide range of temperatures from 310 K to 500 K using direct long-timescale simulations at these temperatures (see Table 1). These solutes have transport rates that are sufficiently large to capture transport events at lower temperatures, allowing us to obtain converged estimates of the rates at physiological temperature. Comparison of these directly determined rate constants with those predicted by fitting c″ and *G*_0_ using four elevated temperature simulations (*T* = 440 K, 460 K, 480 K, & 500 K) shows excellent overall agreement for all four solutes over the entire temperature range, directly validating our method (Fig. 4C). The solutes are suitably different in size, polarity, and chemical composition to suggest that the method is generally applicable to any small-molecule solute. To validate the reliability of the simulations we repeated the simulations for three representative solutes (ethanol, caffeine, and glycerol) that span three order-of-magnitude of permeabilities. Figure S9 shows that the kinetic rate constants reported in this paper are quantitatively reproducible across independent sets of different sampling timescales.

### Calculating solute permeabilities and comparison to experimental measurements

The permeability *P* of a solute diffusing through a membrane patch of area *S* can be obtained from the transport rate *r* observed in a simulation using:

9 $$P=r/(S\cdot C),$$

where *C* is the solute concentration in the aqueous phase (see *Supporting Material* for details). Transport rates determined via extrapolation from high temperature simulations can be obtained using *r* = *k · C*.

Experimentally, solute permeabilities are determined using a transwell assay from the concentrations in the apical (luminal, *C*_L_) and basolateral (abluminal, *C*_A_) compartments after time *t* using:

10 $$P={V}_{{\rm{A}}}\cdot {C}_{{\rm{A}}}/(t\cdot S\cdot {C}_{{\rm{L}}}),$$

where *V*_A_ is the volume of the basolateral compartment (see *Supporting Material* for details).

**Figure 5:**
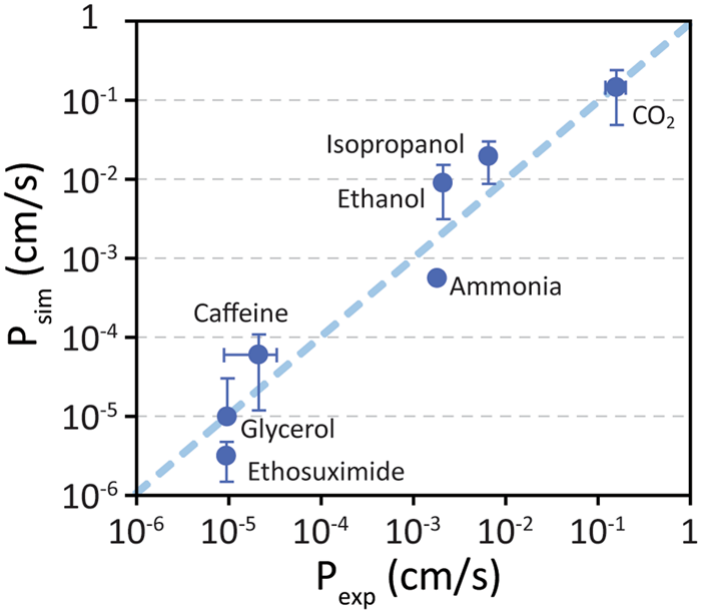
Comparison of predicted and experimentally measured permeabilities. Computationally predicted permeabilities are in excellent agreement with experimentally measured values for molecules spanning nearly five orders of magnitude in permeability. See Table 2 for details of experimental values.

Figure 5 compares the permeabilities obtained from simulations of the seven different solutes chosen for this study with experimentally determined values. The overall quantitative agreement is very good and spans nearly five orders of magnitude in permeability from 3.2 × 10^−6^ cm/s to 0.15 cm/s. The numerical data for this figure are provided in Table 2.

**Table 2:** Comparison of trans-bilayer permeabilities derived from MD simulations (*P*_Sim_) with permeabilities measured using an experimental transwell assay (*P*_app_).

Name	Structure	*P*_app_ (cm/s)	*P*_sim_ (cm/s)
Carbon -dioxide	O=C=O	1.6 ± 0.4 × 10^−1^	1.5 ± 0.1 × 10^−1^
Ethanol		2.1 × 10^−3^	9.3 ± 0.6 × 10^−3^
Isopropanol		6.5 × 10^−3^	2.0±1.1 × 10^−2^
Caffeine		2.1 ± 1.2 × 10^−5^	6.2 ± 3.5 × 10^−5^
Ethosuximide		9.7 × 10^−6^	1.0 ± 2.1 × 10^−5^
Glycerol		9.5 × 10^−6^	3.2 ± 1.7 × 10^−6^
Ammonia	NH_3_	2.7 × 10^−3^	8.10 × 10^−4^

Values for *P*_app_ were obtained from experimental studies. *P*_app_ for CO_2_ was obtained from the rate constant (*k*_m_) for transport across a supported lipid bilayer at room temperature (*P*_app_ = *k*_m_/2) (Gutknecht *et al*.)^39^. *P*_app_ for ethanol and isopropanol were obtained from the rate constants (*k*_m_) for transport into red blood cells (*P*_app_ = *k*_m_/2) (Brahm *et al*.)^40^. *P*_app_ for caffeine was obtained as part of this work from transwell measurements using stem-cell derived hBMECs (see *Supplementary Information* for details). *P*_app_ for ethosuximide was obtained from transwell measurements using MDCKII cells (Summerfield *et al*.)^32^. *P*_app_ for glycerol was obtained from transwell measurements using bovine BMECs (Shah *et al*.)^33^. *P*_app_ for NH_3_ was obtained from basolateral and apical membrane of principal cells (PCs) and intercalated cells (ICs) in perfused rabbit cortical collecting ducts (CCDs)^41^.

### Apical versus basolateral trans-bilayer diffusion

Despite differing lipid composition (Fig. S1), the slope of the Arrhenius plot for the permeation rate constant and temperature dependence of ethanol and caffeine are identical for apical and basolateral hBMEC bilayers (Fig. S10). Both molecules have very different chemical properties and structures, yet still permeate at the same rates in both bilayers, suggesting that knowledge of the permeation rates of either bilayer is sufficient to calculate the overall trans-cellular BBB transport permeability.

## Conclusion

High-temperature simulations dramatically increase the rate of solute transport across lipid bilayers. This allows capturing spontaneous solute transport using unbiased MD simulations. The free energy barrier height was found to vary linearly with temperature, allowing extrapolation of the barrier height to physiological temperatures. However, transport kinetics were found to deviate significantly from Arrhenius behavior. Here we show that this deviation is the result of the change in lipid diffusion with temperature. Correcting for this effect allows accurate prediction of physiological temperature transport kinetics by extrapolation from high-temperature simulations. Comparison of the simulation-predicted solute permeabilities through BBB lipid bilayers to an experimental hBMEC transwell assay shows excellent agreement for a chemically diverse set of solutes that spans nearly five orders of magnitude in permeability, validating the method and suggesting that it is broadly applicable to predict BBB permeabilities.

## Supplementary information

Supplementary Information
