# Supplementary material for: An experimentally validated approach to calculate the blood-brain barrier permeability of small molecules
Source: Sci Rep. 2019 Apr 16;9:6117. doi: 10.1038/s41598-019-42272-0 (PMC6467875; doi:10.1038/s41598-019-42272-0)
Supplement: Supplementary file 1 — Supplementary Information [file 41598_2019_42272_MOESM1_ESM.pdf]

An experimentally validated approach to calculate the blood-brain barrier permeability of small molecules

Yukun Wang[1], Erin Gallagher[1], Christian Jorgensen[1], Evan P. Troendle[2], Dan Hu[3],

Peter C. Searson[1,4,*], & Martin B. Ulmschneider[2,*]

[1]Institute for NanoBioTechnology, Johns Hopkins University, Baltimore, Maryland, USA
[2]Department of Chemistry, King's College, London, UK
[3]Institute of Natural Sciences and Department of Mathematics, Shanghai Jiao-Tong University, Shanghai, China
[4]Department of Materials Science and Engineering, Johns Hopkins University, Baltimore, Maryland, USA

Supplementary Information

Calculating solute diffusion coefficients along the membrane normal

Solute diffusivities are calculated using the method of Hummer and coworkers[1]. Each solute was placed near the free energy barrier maximum (position G_B) and held fixed using a harmonic umbrella potential with a spring constant 100 kJ mol^{-1} nm^{-2}. The temperature was varied between 310K and 500K. The diffusion coefficient D_z was calculated from these simulations using an autocorrelation function:

$$D_Z = \frac{\langle z_{COM}{}^2\rangle - \langle z_{COM}\rangle^2}{\tau} \;, \qquad \text{(Eq. S1)}$$

where τ was calculated from the autocorrelation function:

$$\tau = \int_0^{\infty} \frac{\langle \delta z_{COM}(t)\delta z_{COM}(0)\rangle}{\langle z_{COM}{}^2\rangle - \langle z_{COM}\rangle^2} dt \;, \qquad \text{(Eq. S2)}$$

With

$$\delta z_{COM}(t) = z_{COM}(t) - \langle z_{COM}\rangle. \qquad \text{(Eq. S3)}$$

The first 10% of the trajectory was excluded from the calculations to allow for the system to equilibrate. Variances were calculated by dividing the simulation into 3 equal parts and calculating the diffusion coefficient for each part.

Calculating lipid diffusion coefficients in the plane of the membrane

When calculating the lipid diffusion coefficient, a number of methodological issues need to be addressed, including the treatment of the periodic boundary conditions as well as finite-size effects of the box.[2–4] Therefore, the lateral lipid diffusion coefficient was determined from the mean square displacement of phosphorus atoms with respect to unwrapped periodic boundary coordinates in the plane of the membrane (xy-plane). Since the lipid composition of the leaflet is the same on average, the lateral diffusion coefficient,

$$D_{Lipid} = \frac{\langle r(t)\rangle^2}{4t} \qquad \text{(Eq. S4)}$$

was calculated by averaging the mean square displacement $<r(t)>^2$ observed in both bilayer leaflets. Cholesterol within the bilayer are excluded due to their susceptibility to flip-flop.

Calculating membrane permeabilities from experiments and simulations

The permeability P (cm/s) is related to the net flux J (#/cm^2·s) through a membrane patch by:

$$J = P \cdot (C_o - C_i), \qquad \text{(Eq. S5)}$$

where C_i (#/m^3) and C_o are the concentrations on either side of the membrane.

Experiments measure changes in concentration on either side of the membrane, which corresponds to measuring the net flux across the membrane (i.e. forward minus backward).

In the simulations each particle can be traced and we can obtain the backward and forward fluxes separately. If the particles do not interact, which is checked in the simulations, the concentration can be thought of to be constant on one side of the bilayer and zero on the other. The permeability is the calculated from the averaged the total forward and backward fluxes through the bilayer.

Due to pressure coupling the box volume and area of the bilayer patch, *S*, will vary during the simulation and need to be averaged. The flux is calculated by counting the total number of forward and backward permeation events through the bilayer, dividing by the simulation length and averaging the result:

$$J = (r_i + r_o) / S, \qquad \text{(Eq. S6)}$$

where r_i (#/s) and r_o are the forward and backward transport rates, respectively. The permeability is then obtained using $r = P \cdot S \cdot C$:

$$P = (\#_i + \#_o) / (2 \cdot t \cdot S \cdot C), \qquad \text{(Eq. S7)}$$

where # are the number of transport events, *t* is the simulation time, and *S* and *C* are simulation averages of the membrane area and concentration (the volume changes), respectively.

TABLE S1

	NH_3	CO_2	Ethanol	Isopropanol	Glycerol	Caffeine	Ethosuximide
**MW [Da]**	17	44	46	60	92	194	141
**Dipole moment [D]**	1.47	0	1.69	1.58	2.56	3.65	-
**Log P***	-	0.83	-0.3	0.59	-1.76	-0.1	0.38
**HB acceptors [#]**	1	0	2	2	6	5	2
**HB donors [#]**	3	0	1	1	3	0	1

Table S1 | Molecular properties of the solute molecules studied here. *Octanol-water partition coefficient, log P. The log P values for NH_3, CO_2, ethanol, isopropanol and glycerol are from experiment (Hansch et al. 2005).[5] The value for ethosuximide is from experiment (Atkinson, Berg, 1988).[6]

TABLE S2

$\Delta G^{\ddagger}$	NH_3	CO_2	Ethanol	Isopropanol	Glycerol	Caffeine	Ethosuximide
**Slope, *g***	-0.015	-0.0047	-0.011	-0.012	-0.022	-0.020	-0.0164
**Intercept, G_0**	10.9	3.3	7.0	6.8	15.7	12.3	11
**R^2**	0.992	0.881	0.990	0.991	0.997	0.988	0.998

D_Z	NH_3	Ethanol	Caffeine	Cholesterol
**Slope, m_D**	1	1	1	1
**Intercept, *b***	3.6	2.8	2.0	1.2
**R^2**				

l_b	NH_3	CO_2	Ethanol	Isopropanol	Caffeine
**Slope, m_l**	2	2	2	2	2
**Intercept, *a***	-11.60	-11.31	11.36	-11.34	-12.14
**R^2**					

Table S2 | Fitting data. $\Delta G^{\ddagger}$: Linear fit of the main barrier height with respect to temperature. The main barrier height of all solutes deceases linearly with increasing temperature (see Figure 1D) and can be fit to: $\Delta G^{\ddagger}(T) = g \cdot T + G_0$, with an average R^2 of 0.98. **D_Z:** Diffusion coefficient along the membrane normal was fit against the lipid diffusion coefficient using: $\ln(D_Z) = \ln(b) + m_D \cdot \ln(D_L)$, with parameters *m* and *b* (c.f. Figure 4). **l_b :** Barrier width was fit against temperature using: $\ln(l_b) = \ln(a) + m_l \cdot \ln(T)$, with fitting parameters *m* and *a* (c.f. Figure 4).

TABLE S3

T[K]	310	330	350	380	400	420	440	460	480	500
**Simulation length [µs]**	10	3	2	1	1.6	1.6	0.2	0.2	0.2	0.2
**D_{Apical} [µm^2/s]**	0.6±0.1	3.7±0.2	11±0.3	31±3	77±5	103±7	124±6	189±11	258±30	401±44

Table S3 | Temperature dependence of the lipid diffusion coefficient of the apical hMBEC bilayer. Average lateral (i.e. in-plane) lipid diffusion coefficient (D) calculated from apical lipid bilayer simulations with ethanol as a solute. There are 96 lipid molecules in the system, the composition is shown in Figure S2. The phosphorus atoms of the lipids were chosen to calculate the average mean-squared displacement.

TABLE S4

	Ethanol		Caffeine‡		Glycerol‡		Ethanol reproduction		Caffeine reproduction		Glycerol reproduction	
T [K]	t [µs]	N_{trans} [#]	t [µs]	t [µs]	N_{trans} [#]	N_{trans} [#]	t [µs]	N_{trans} [#]	t [µs]	N_{trans} [#]	t [µs]	N_{trans} [#]
310[a]	10	11	-	-	-	-	2.0	7	-	-	-	-
330[a]	3.0	25	8.6	1	-	-	-	-	-	-	-	-
350[a]	2.0	76	-	-	-	-	-	-	-	-	-	-
350[b]	1.3	47	-	-	-	-	-	-	-	-	-	-
360[a]	-	-	4.0	15	-	-	-	-	-	-	-	-
380[a]	1.0	188	4.0	33	-	-	-	-	-	-	-	-
380[b]	0.6	118	-	-	-	-	-	-	-	-	-	-
400[a]	0.6	244	1.4	46	-	-	-	-	-	-	-	-
400[b]	0.5	209	1.0	30	-	-	-	-	-	-	-	-
420[a]	0.2	159	1.0	85	-	-	0.38	321	-	-	-	-
420[b]	-	-	1.0	69	-	-	-	-	-	-	-	-
440[a]	0.2	323	1.0	147	2.0	19	0.42	629	0.6	46	2.0	10
440[b]	-	-	1.0	177	-	-	-	-	-	-	-	-
460[a]	0.2	474	0.32	120	2.0	50	-	-	0.32	107	2.0	54
480[a]	0.2	816	0.32	253	2.0	129	-	-	0.32	216	2.0	101
500[a]	0.2	1375	0.32	611	1.0	130	1.0	5282	0.32	386	1.0	272

Table S4 | Summary of the unbiased simulations listing the simulation length (*t*) and number of spontaneous transitions (N_{trans}) for reproduction set.

Figure S1

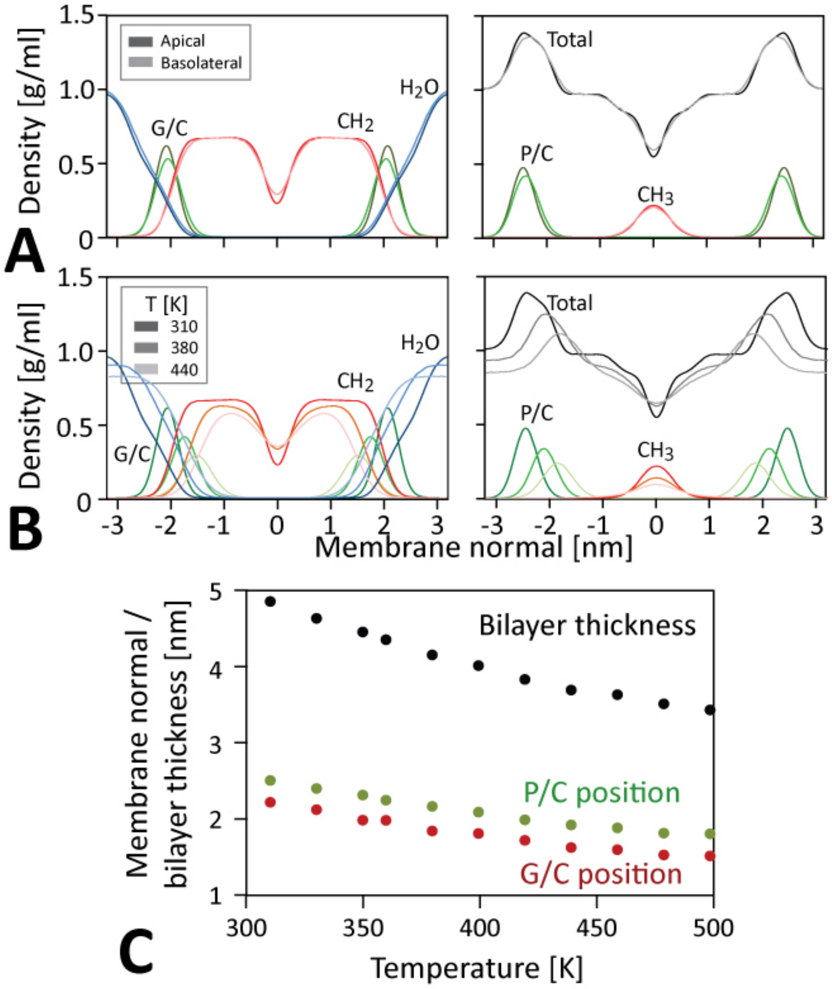

Figure S1 | Trans-bilayer density profile of the key structural groups of hBMEC bilayers. A: Apical and basolateral bilayers have very similar cross-sectional density profiles and the positions of the principal structural groups (CH_3 = methyl, CH_2 = acyl tails, P/C = phosphocholine headgroups, G/C = glycerol-carbonyl linker, H_2O = water) along the membrane group are identical. The density profiles are averages over 10 µs. **B:** The effect of heating on the lipid bilayer can be visualized by plotting the equilibrium trans-bilayer density profiles for temperatures in the range 37–167 °C (dark to light colours). Comparison of the principal structural groups shows temperature induced broadening of the Gaussians and a monotonic shift of the positions associated with overall broadening of the membrane. However, the overall chemical profile does not change qualitatively and the change is highly predictable. **C:** Temperature dependence of the position along the membrane normal (z-axis) of the P/C and G/C groups, which serve as an indicator of membrane thickness (defined as average distance between the phosphates in the upper and lower bilayer leaflet) of the apical bilayer. Both the bilayer thickness decreases monotonically and predictably with increasing temperature.

Figure S2

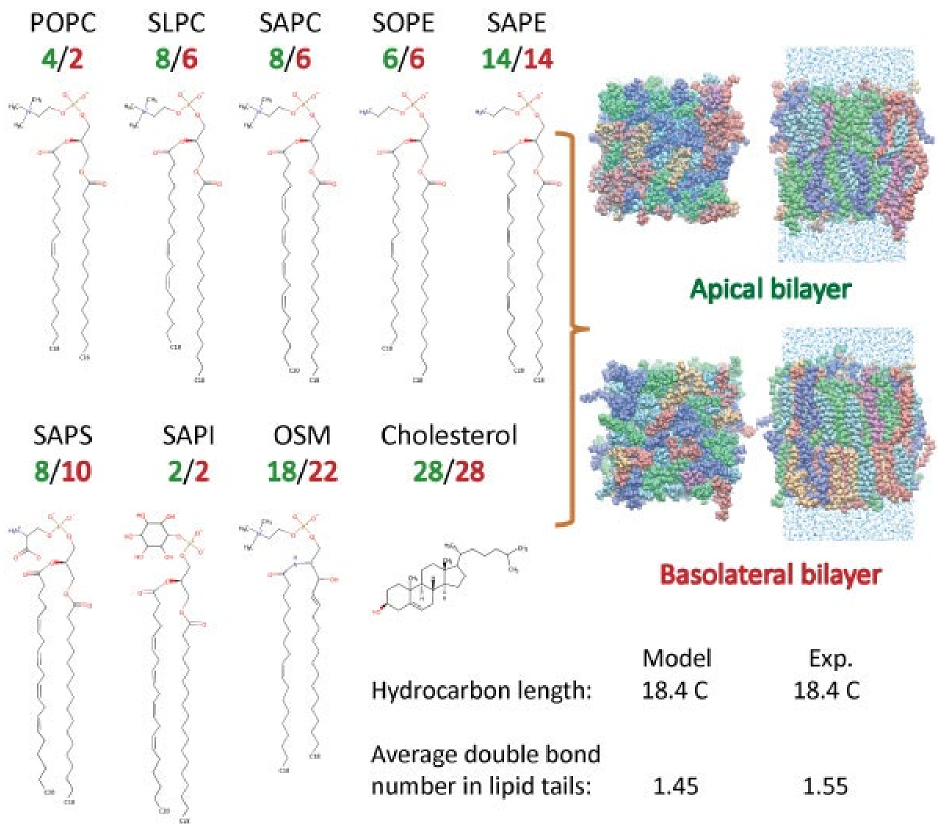

Figure S2 | Atomic detail models of the apical and basolateral hBMEC bilayers. Each bilayer consists of 9 lipids: POPC, SLPC, SAPC (red); SOPE, SAPE (blue); SAPS (orange); SAPI (purple); OSM (green); and cholesterol (cyan). The number of each lipid species in the molecular model of the apical and basolateral bilayer membrane of hBMECs is indicated. Atomic detail models were constructed using the CHARMM-GUI membrane builder[7–12]. Each bilayer was equilibrated for 100 ns.

Figure S3

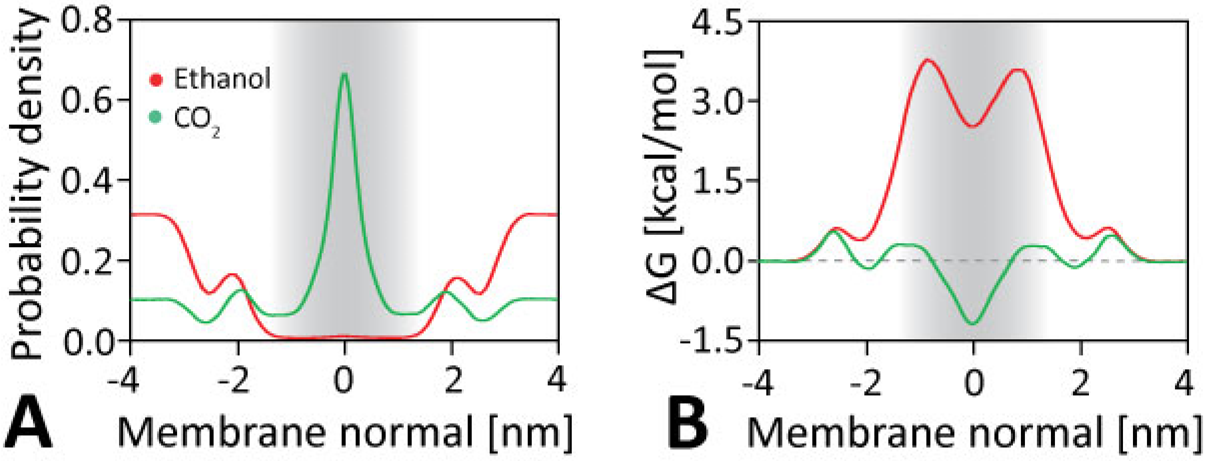

Figure S3 | Derivation of the transbilayer free energy profile from the solute density. Trans-bilayer free energy profiles (**B**) are calculated from the time-averaged solute density (**A**) along the membrane normal obtained from unbiased atomic detail MD equilibrium simulations for CO_2 and ethanol at 310K.

Figure S4

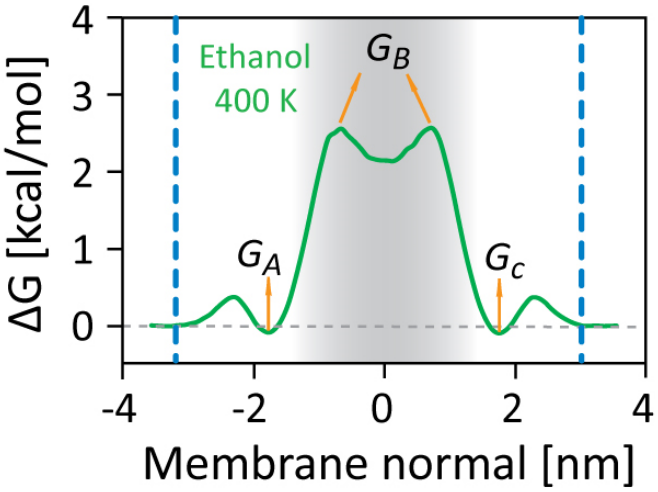

Figure S4 | Trans-bilayer free energy profile for ethanol at 380K in pure POPC. A. G_A and G_C indicate the two free energy minima located at the membrane-water interface, while G_B represents the barrier height. If the central barrier has a saddle shape with two peaks, as in the present profile for ethanol, the barrier height G_B is calculated as the averaged height of both barriers. The dashed lines indicate the planes solutes need to cross consecutively in order to record a transport event. The planes are positioned just outside the bilayer in the aqueous phase, since for hydrophobic molecules such as CO_2 desorbing from bilayer is the chief permeation barrier.

Figure S5

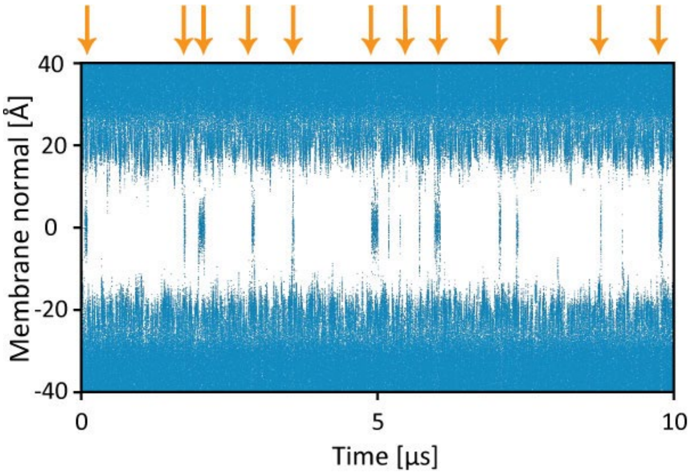

Figure S5 | Ethanol permeation through the hBMEC apical bilayer at 310K. Plotting the position of individual ethanol molecules with respect to the membrane centre of mass reveals several transport events (marked with arrows). Solute flux was calculated by tracking the crossing of permeating molecules through planes perpendicular to the bilayer normal. Note that the transport rate across the hydrophobic core is not necessarily equal to the total rate, as some molecules have significant barriers for partitioning out of the membrane interface (c.f. Figure S4 & Figure S6).

Figure S6

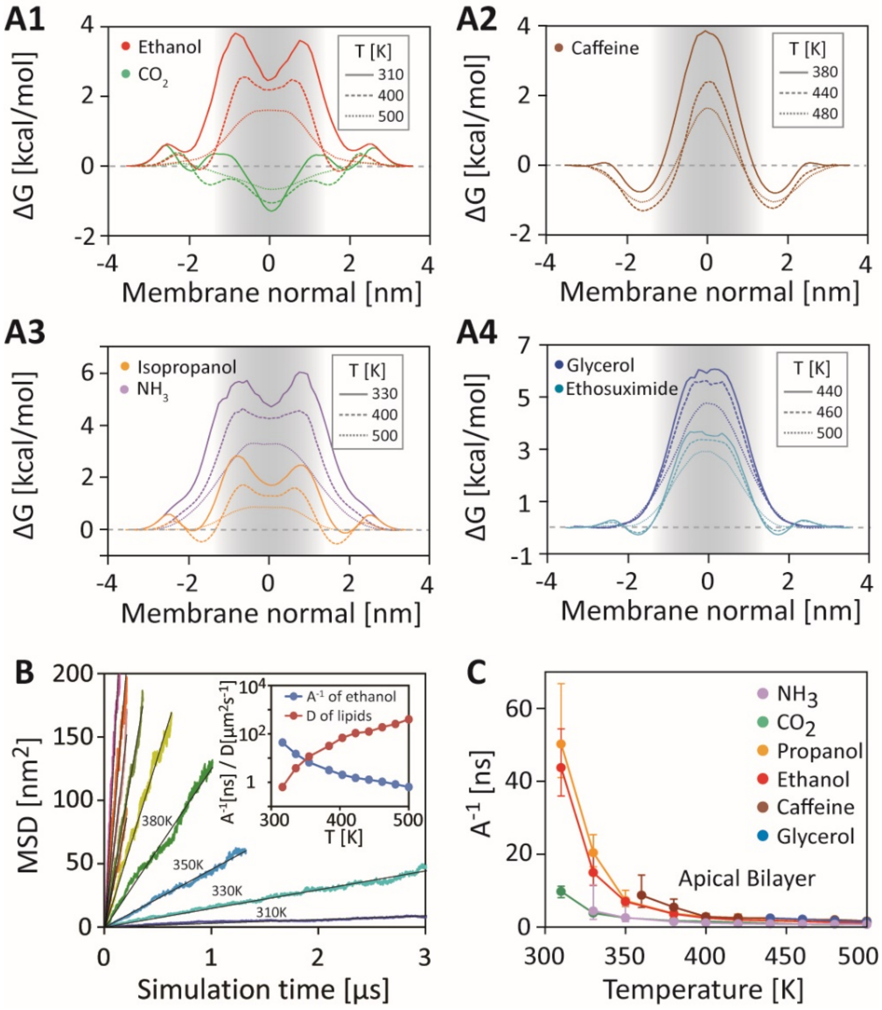

Figure S6 | Temperature dependence of the trans-bilayer free energy profiles. A1-A4. Free energy profiles calculated from unbiased atomic detail MD equilibrium simulations for each of the compounds studied here at three temperature points. Increasing the temperature generally flattens the free energy profile and lowers the height of the transmembrane free energy barrier. **B.** The lipid diffusion coefficient, D_L, can be derived from the mean squared displacement (MSD) of the diffusing lipid at different temperatures. The inset shows that the lipid diffusion coefficient D_L increases rapidly with increasing temperature, while the pre-factor *A* of ethanol molecules decreases rapidly with increasing temperature. **C.** Pre-factor A for six solutes in apical hBMEC lipid bilayers calculated by an Arrhenius relation. Inverse of pre-factor, A^{-1}, decreases with increasing temperature; this behavior can be divided into two zones: a fast-changing region (T < 400K) and slowly-changing region (T > 400K).

Figure S7

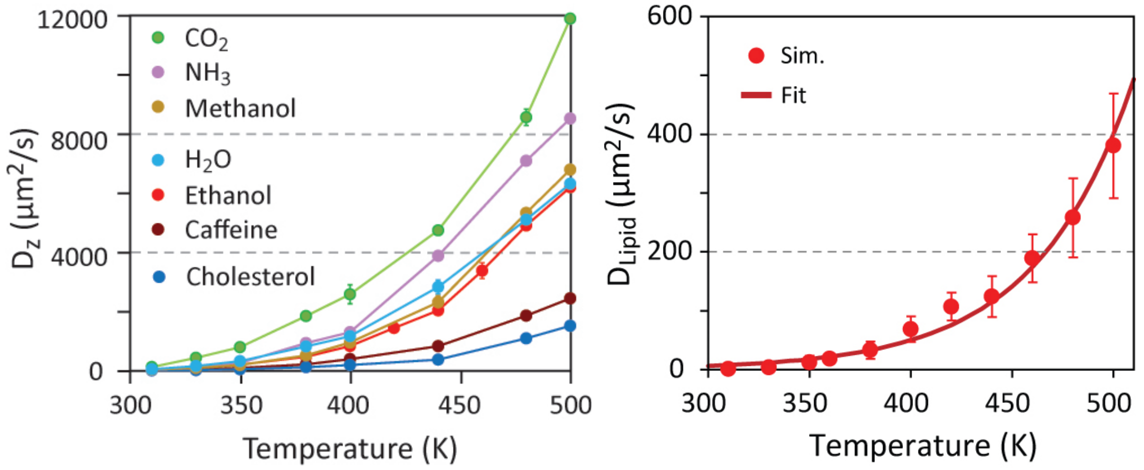

Figure S7 | Temperature dependence of the solute and lipid diffusion coefficients in the apical lipid bilayer. A. Solute diffusivities along the membrane normal were calculated using the method of Hummer and co-workers[1]. Each solute was restrained to the region of the free energy barrier maximum by applying a harmonic potential. The temperature dependent solute diffusion coefficient along the membrane normal, $D_z(T)$, was calculated from these simulations using an autocorrelation function. **B.** The average lipid diffusion coefficient in the plane of the membrane, $D_L(T)$, was calculated by averaging the diffusion of the phosphate groups for all lipid species in the bilayer. The data can be fit to the empirical function: $y = a \exp(b \cdot (T - T_0))$, a = 33, b = 0.0208, T_0 = 380, in the range 300-500 K.

Figure S8

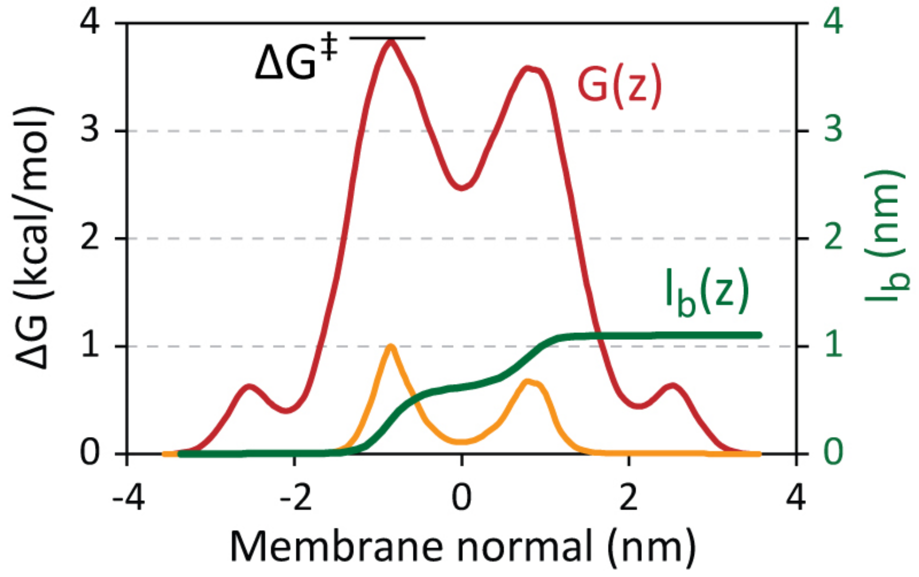

Figure S8 | Calculation of the free-energy barrier width *l_b*. The barrier width l_b (green) is calculated from the free energy profile G(z) (red) using equation 3. The figure shows that the chief contributions to the integral (yellow) are near the barrier peaks.

Figure S9

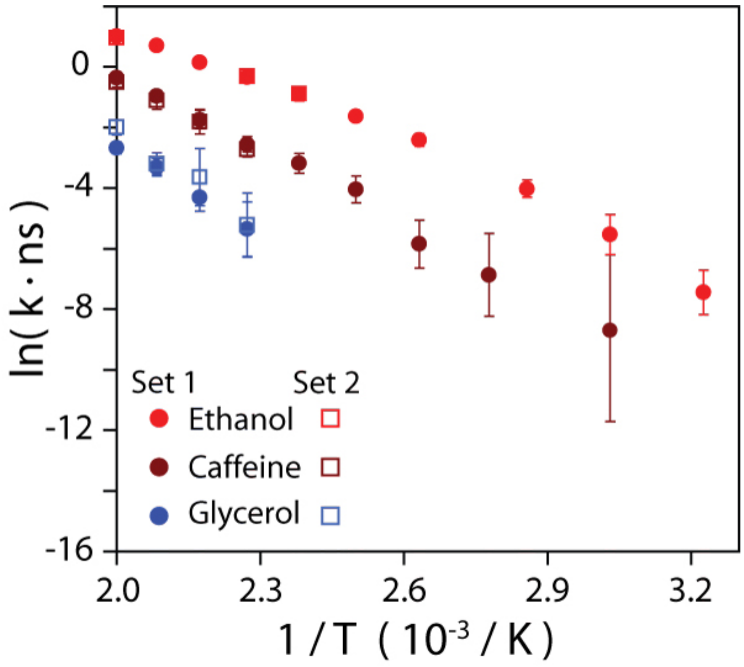

Figure S9 | Comparison of the kinetics obtained from two different set of simulations. Two independent sets of simulations (set 1, 50.9 µs; set 2, 9.8 µs) of ethanol, caffeine, and glycerol show excellent quantitative overlap over a broad range of temperatures.

Figure S10

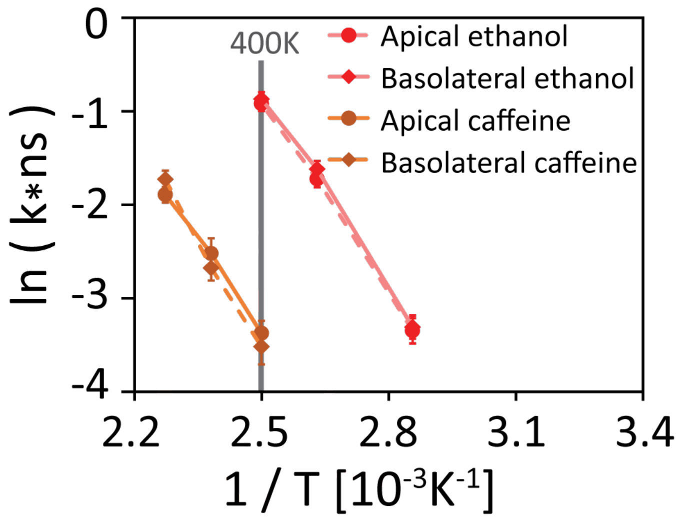

Figure S10 | The permeability of the apical and basolateral hBMEC bilayers is identical. Arrhenius plots of the transport kinetics obtained from apical and basolateral hBMEC bilayers at different temperatures for ethanol and caffeine are quantitatively identical within the errors of the calculations.

References

1. Hummer, G. Position-dependent diffusion coefficients and free energies from Bayesian analysis of equilibrium and replica molecular dynamics simulations. *New J. Phys.* **7**, 34 (2005).
2. Anézo, C., de Vries, A. H., Höltje, H.-D., Tieleman, D. P. & Marrink, S.-J. Methodological issues in lipid bilayer simulations. *J. Phys. Chem. B* **107**, 9424–9433 (2003).
3. Vögele, M., Köfinger, J. & Hummer, G. Hydrodynamics of Diffusion in Lipid Membrane Simulations. *Phys. Rev. Lett.* **120**, 268104 (2018).
4. Matsumoto, K., Kusaka, J., Nishibori, A. & Hara, H. Lipid domains in bacterial membranes. *Mol. Microbiol.* **61**, 1110–1117 (2006).
5. Hansch, C., Leo, A. & Hoekman, D. H. *Exploring QSAR: fundamentals and applications in chemistry and biology*. **557**, (American Chemical Society Washington, DC, 1995).
6. Atkinson, H. C. & Begg, E. J. The binding of drugs to major human milk whey proteins. *Br. J. Clin. Pharmacol.* **26**, 107–109 (1988).
7. Brooks, B. R. *et al.* CHARMM: The Biomolecular Simulation Program. *J. Comput. Chem.* **30**, 1545–1614 (2009).
8. Jo, S., Kim, T. & Im, W. Automated Builder and Database of Protein/Membrane Complexes for Molecular Dynamics Simulations. *PLOS ONE* **2**, e880 (2007).
9. Jo, S., Kim, T., Iyer, V. G. & Im, W. CHARMM-GUI: A web-based graphical user interface for CHARMM. *J. Comput. Chem.* **29**, 1859–1865 (2008).
10. Jo, S., Lim, J. B., Klauda, J. B. & Im, W. CHARMM-GUI Membrane Builder for Mixed Bilayers and Its Application to Yeast Membranes. *Biophys. J.* **97**, 50–58 (2009).
11. Wu, E. L. *et al.* CHARMM-GUI Membrane Builder toward realistic biological membrane simulations. *J. Comput. Chem.* **35**, 1997–2004 (2014).
12. Lee, J. *et al.* CHARMM-GUI Membrane Builder for Complex Biological Membrane Simulations with Glycolipids and Lipoglycans. *J. Chem. Theory Comput.* **15**, 775–786 (2019).
